# Association of ABO polymorphisms and pancreatic Cancer/ Cardiocerebrovascular disease: a meta-analysis

**DOI:** 10.1186/s12881-020-0975-8

**Published:** 2020-02-24

**Authors:** Yanxia Li, Luyang Liu, Yubei Huang, Hong Zheng, Lian Li

**Affiliations:** Department of Epidemiology and Biostatistics, National Clinical Research Center for Cancer, Key Laboratory of Molecular Cancer Epidemiology of Tianjin, Tianjin Medical University Cancer Institute and Hospital, Tianjin, China

**Keywords:** ABO gene polymorphism, rs505922, rs657152, Cardiocerebrovascular diseases, Cancer, Meta-analysis

## Abstract

**Background:**

ABO gene polymorphisms have been reported to be associated with the risk of multiple cancers and cardiocerebrovascular diseases. However, the results remained controversial. In this study, we conducted a systematic review and meta-analysis to clarify the association between two SNPs (rs505922 and rs657152) in ABO gene and cancers/cardiocerebrovascular diseases.

**Method:**

All eligible case-control studies come from PubMed, Embase and Web of Science up to Jan. 1, 2019. Pooled odds ratios (ORs) with 95% confidence intervals (CIs) were used to assess the corresponding associations. Sensitivity analysis, publication bias assessment, and heterogeneity test were performed using STATA 12.0.

**Results:**

A total of nineteen articles involving twenty-two case-control populations were included according to inclusion and exclusion criteria. Twelve populations (20,820 cases and 27,837 controls) were used to evaluate the relationship between rs505922 and overall cancers and nine populations (22,275 cases and 71,549 controls) were included to assess the association between rs505922 and cardiocerebrovascular diseases. The results showed a significant association between the rs505922 polymorphism and cancers (CvsT: OR = 1.13, 95%CI = 1.05–1.22, *P* = 0.001), and cardiocerebrovascular diseases (OR = 1.36, 95%CI = 1.19–1.57, *P* < 0.001). Five populations (8660 cases and 10,618 controls) were included to evaluate association between rs657152 and cancers and five populations (8105 cases and 6712 controls) were included to estimate the relationship between rs657152 and cardiocerebrovascular diseases. The result of meta-analysis reveals that rs657152 was significantly associated with cancers (OR = 1.18, 95%CI = 1.13–1.23, *P* < 0.001) and cardiocerebrovascular diseases (OR = 1.54, 95%CI = 1.24–1.92, *P* < 0.001).

**Conclusion:**

Our study suggested that ABO polymorphisms might serve as a risk factor of pancreatic cancers and cardiocerebrovascular diseases.

## Background

The incidence of cancer and cardiovascular disease increases with age and both of them are related to inflammation and thrombosis et al. [1, 2]. Epidemiological studies have demonstrated that ABO blood groups were associated with several chronic inflammation related diseases, including cancers and cardiocerebrovascluar diseases [3–5]. Although a possible common pathogenic mechanism involving the von Willebrand factor between ABO blood group system and cancer/cardiovascular disease has been discussed [6], the relationship between histoblood group antigens and inflammation related diseases remains unknown and the regulatory mechanisms underlying ABO expression was still unclear.

The ABO blood group system is composed of complex carbohydrate structures that are biosynthesized by A- and B- transferases encoded by ABO gene. ABO gene is located at the 9q34 region of the chromosome and encodes enzyme glycosyltransferase with two main allele (A and B), a specific glycosyltransferase catalyzes the covalent attachment of N-acetylgalactosamine or D-galactose to a common precursor side chain (H determinant), ultimately to an A or B antigen [7]. Several SNPs in the ABO gene have been suggested to be associated with increased risks to the development of cancer and cardiocerebrovascluar disease by genome-wide association studies (GWAS) and candidate gene studies [8–11]. Particularly, the most widely investigated SNPs of the ABO gene were rs505922 and rs657152 [12–15]. Meta-analysis that evaluated the relationship between rs505922 SNP and overall cancer have already been reported by Duan et al. [16] four years ago. However, two new studies of cancer risk [11, 17] and several studies of cardiocerebrovascluar diseases risk [14, 15, 18–21] have been reported in recent years and the relationship between SNPs in ABO gene and cancer/cardiocerebrovascular diseases risk was still unclear. Here, we have performed a meta-analysis including the newly published studies and evaluated the associations between polymorphisms of ABO gene and cancers/cardiocerebrovascular diseases.

## Methods

### Search strategy

Two investigators (Yanxia Li and Luyang Liu) performed a systematic literature search in three databases: PubMed, Web of Science and Embase to identify relevant articles published from the initial to Jan 1, 2019. The following search terms were used either separately or in combination: “SNP” “Polymorphism” and “Polymorphism, Single Nucleotide” “rs505922, rs657152” and “neoplasms” “carcinoma” “tumor” “cancer” and “vascular diasease” “cardiovascular disease” “cardiac-cerebral disease” and “ABO Blood-Group System” “Lewis Blood-Group System” “ABO”. Once suitable studies were singled out from the search results, other potentially relevant articles were identified by cross-references within eligible studies. The references of each identified articles were also searched manually to identify eligible studies.

### Selection criteria

The inclusion criteria for these studies were as follows: 1) Studies evaluating the association between rs505922/rs657152 variants and cancers or cardiocerebrovascular diseases; 2) The study design was a case-control study in humans (both hospital based case-control study and cohort based nested case-control study were included); 3) Researches containing universal allele and genotype data; 4) Studies written in English. The exclusion criteria were as follows: 1) Studies did not describe the association of ABO gene polymorphism with cancers or cardiocerebrovascular diseases; 2) Systematic reviews or articles focusing on animals; 3) Studies that did not provide usable data for meta-analysis.

### Data extraction and quality assessment

Two investigators (Yanxia Li and Luyang Liu) independently extract data and verify the accuracy of the data. The following information was extracted from each article: first authors, publication years, ethnicity, cancer types, and study design (hospital- or population-based), sample size of subject, number of cases and controls for each genotype, OR and 95% CI in allele model. When there was no data of allele model in the article, we calculated the data of allele model by using the formula. To assess the quality of the study, we used the Newcastle-Ottawa Scale (NOS) with a nine-star system [22]; this scale assesses the quality of cohort and case-control studies. The highest score of NOS is nine stars: four stars for the selection process, three stars for exposure/outcome, and two stars for comparability. A score of seven or above was considered to be high-quality study.

### Statistical analysis

We used odds ratios (ORs) and 95% confidence intervals (CIs) to assess the relationship between each SNP and the risk of cancers or cardiocerebrovascular diseases. The association was examined using allele model. The significance of the pooled OR was determined by the *Z*-test. Subgroup analyses were conducted according to cancer types, ethnic groups and sources of controls. Heterogeneity between articles was identified with *Q*-test and *I*^*2*^ index, *I*^*2*^ values of > 50% indicated heterogeneity among studies. If there was heterogeneity (*I*^*2*^ > 50%) between studies, we used a random effect model (DerSimonian-Laird method), otherwise we used a fixed effect model (Mantel-Haenszel method). Sensitivity analysis was performed to assess the effects of individual studies on pooled results and the stability of the results. We used Egger’s and Begg’s test to evaluate the publication bias, with a *P* > 0.05 considered to be evidence for no potential publication bias. Trim and fill method was also applied in detecting publication bias. All the tests were two-sided and *P* < 0.05 was considered to be statistically significant. Stata version 12.0 SE was applied to carry out the statistical analysis.

## Results

### Study characteristics

**Fig. 1:**
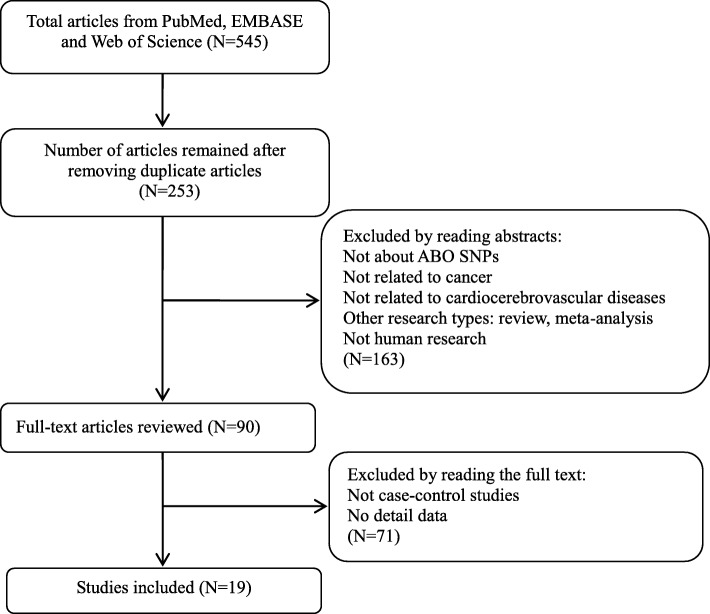
Flow chart of the study selection process

After retrieving the database, a total of 545 records [PubMed (*n* = 146), Web of Science (*n* = 149), EMBASE (*n* = 250)] were obtained. According to the inclusion/exclusion criteria, nineteen articles were included and the detailed flowchart of study selection process was presented in Fig. 1. Twelve studies reported the association between rs505922 and cancer risk [11–13, 17, 23–30] and five studies reported the association between rs657152 and cancer risk [12, 13, 17, 30, 31]. Six studies included nine populations reported the association of cardiocerebrovascular diseases with rs505922 [14, 15, 18–21], and three studies with rs657152 [14, 15, 20]. Detailed characteristics and genotype distribution of included articles for two SNPs were shown in Table 1 and Table 2. In addition, seven additional SNPs which had strong linkage disequilibrium (LD) with rs525922 were reported to be associated with cardiocerebrovascular disease (Table S1) [8, 14, 15, 21, 32–35]. However, the association between these SNPs with cancer risk has not been reported. According to the source of control groups, nine studies were population-based (PB), six studies were hospital-based (HB), and three studies were population and hospital-based(PB/HB) control. For SNP rs505922, ten studies were from Caucasian population [11, 13, 15, 19, 20, 23–26, 29], four studies were from Asian [12, 18, 21, 27] and four studies were from Mixed and African [14, 17, 28, 30]. For the rs657152 polymorphism, there were four studies originating from Caucasian [13, 15, 20, 31], and four studies were from Asian and African or Mixed population [12, 14, 17, 30]. Each study was scored based on the Newcastle-Ottawa Scale (NOS) and detailed study qualities were presented in Table S2 and Table S3.

**Table 1:** Characteristics of the studies included in the meta-analysis of the association between SNPs and cancers

SNP loci	Year	First author	Ethnicity	Cancer/disease	Control^a^	Sample size (case/control)	Case			Control			OR(95%CI)
							Hom^†^ wild	Het^†^	Hom variant	Hom^†^ wild	Het^†^	Hom variant	
rs505922	2015	SC. Markt	Caucasian	Prostate cancer	–	2774/4443	1172	1226	376	1772	2070	601	0.95(0.89–1.02)
	2015	E. Duell	Caucasian	Gastric cancer	PB	365/1284	137	172	49	533	541	188	1.06(0.89–1.26)
	2014	H. Xu	Asian	Pancreatic cancer	PB	256/548	69	124	63	186	246	115	1.24(1.00–1.53)
	2013	C.Rizzato	Caucasian	Pancreatic cancer	HB/PB	1028/2257	342	518	163	860	1055	336	1.13(1.01–1.26)
	2012	E.Poole	Caucasian	Ovarian cancer	HB/PB	5233/6838	2222	2407	603	2987	3044	806	1.02(0.97–1.08)
	2012	D. Li	Mixed	Pancreatic cancer	HB	3851/3934	–	–	–	–	–	–	1.21(1.13–1.30)
	2012	J. Willis	Caucasian	Pancreatic cancer	HB	385/149	–	–	–	–	–	–	1.65(1.20–2.26)
	2012	M.Gates	Caucasian	Breast cancer	PB	1138/1090	489	505	144	471	487	132	1.02(0.90–1.15)
	2011	M.Krawczyk	Caucasian	Cholangiocarcinoma	PB	180/350	84	68	28	154	146	50	0.97(0.74–1.27)
	2011	M. Nakao	Asian	Pancreatic cancer	HB	185/1465	38	101	46	428	745	292	1.31(1.06–1.63)
	2010	B.Wolpin	Mixed	Pancreatic cancer	PB	1534/1583	511	752	271	657	705	221	1.28(1.16–1.42)
	2009	L.Amundadottir	Mixed	Pancreatic cancer	HB/PB	3891/3932	1436	1856	599	1667	1785	480	1.20(1.13–1.29)
rs657152	2015	E. Duell	Caucasian	Gastric cancer	PB	365/1284	–	–	–	–	–	–	1.05(0.89–1.23)
	2014	H. Xu	Asian	Pancreatic cancer	PB	256/548	71	124	61	199	240	108	1.29(1.05–1.60)
	2012	D. Li	Caucasian	Pancreatic cancer	HB	3851/3934	–	–	–	–	–	–	1.19(1.12–1.27)
	2011	C. Rizzato	Caucasian	Pancreatic cancer	PB	686/1255	199	357	130	437	591	227	1.15(1.00–1.31)
	2009	L.Amundadottir	Mixed	Pancreatic cancer	HB/PB	3502/3597	1191	1702	609	1395	1691	511	1.18(1.13–1.23)

^a^*PB* population based control, *HB* hospital based control; †*Hom* homozygous, *Het* heterozygote

**Table 2:** Characteristics of the studies included in the meta-analysis of the association between SNPs and cardiocerebrovascular diseases

SNP loci	Year	First author	Ethnicity	Cancer/disease	Control^a^	Sample size (case-control)	Case			Control			OR(95%CI)
							Hom^†^ wild	Het^†^	Hom variant	Hom^†^ wild	Het^†^	Hom variant	
rs505922	2017	H. Li	Asian	Ischemic stroke	PB	991/1002	511	406	74	657	306	39	1.64(1.41–1.90)
	2017	H. Zhang	Asian	Large artery atherosclerotic stroke	PB	644/642	146	325	173	129	322	191	0.90(0.77–1.05)
	2016	W. Hernandez	African	Venous Thrombosis	HB	146 /432	–	–	–	–	–	–	1.52(1.20–2.00)
	2013	FM.Williams-a^‡^	Caucasian	Ischemic stroke	HB	4092/8383	–	–	–	–	–	–	1.06(1.01–1.14)
	2013	FM.Williams-b^‡^	Caucasian	Ischemic stroke	HB	8443 /54810	–	–	–	–	–	–	1.07(1.03–1.11)
	2011	MP. Reilly	Caucasian	Myocardial infarction	HB	5783/3644	–	–	–	–	–	–	1.20(1.13–1.28)
	2009	DA.Trégouet-a^§^	Caucasian	Venous thromboembolism	PB	419/1228	97	209	113	519	559	150	2.01(1.71–2.35)
	2009	DA.Trégouet-b^§^	Caucasian	Venous thromboembolism	PB	1150/801	299	575	276	339	364	98	1.79(1.57–2.04)
	2009	DA.Trégouet-c^§^	Caucasian	Venous thromboembolism	PB	607/607	177	302	128	265	272	70	1.66(1.41–1.95)
rs657152	2016	W. Hernandez	African	Venous Thrombosis	HB	146 /432	–	–	–	–	–	–	1.39(1.10–1.80)
	2011	MP. Reilly	Caucasian	Myocardial infarction	HB	5783/3644	–	–	–	–	–	–	1.19(1.12–1.27)
	2009	DA.Trégouet-a^§^	Caucasian	Venous thromboembolism	PB	419/1228	89	208	122	472	579	177	1.91(1.63–2.24)
	2009	DA.Trégouet-b^§^	Caucasian	Venous thromboembolism	PB	1150/801	276	575	299	318	373	110	1.77(1.55–2.02)
	2009	DA.Trégouet-c^§^	Caucasian	Venous thromboembolism	PB	607/607	171	302	134	249	280	78	1.58(1.34–1.86)

^a^*PB* population based control, *HB* hospital based control; †*Hom* homozygous, *Het* heterozygote; a^‡^ represent the MOnica Risk, Genetics, Archiving and Monograph(MORGAM) and the Wellcome Trust Case Control Consortium 2(WTCCC2) population, b^‡^ represent MetaStroke population; a^§^ represent GWAS population from 4 different French centers, b^§^represent MARseille THrombosis Association study (MARTHA) population, c^§^represent FARIVE that is a multicenter case-control study

### Meta-analysis of rs505922 polymorphism

**Fig. 2:**
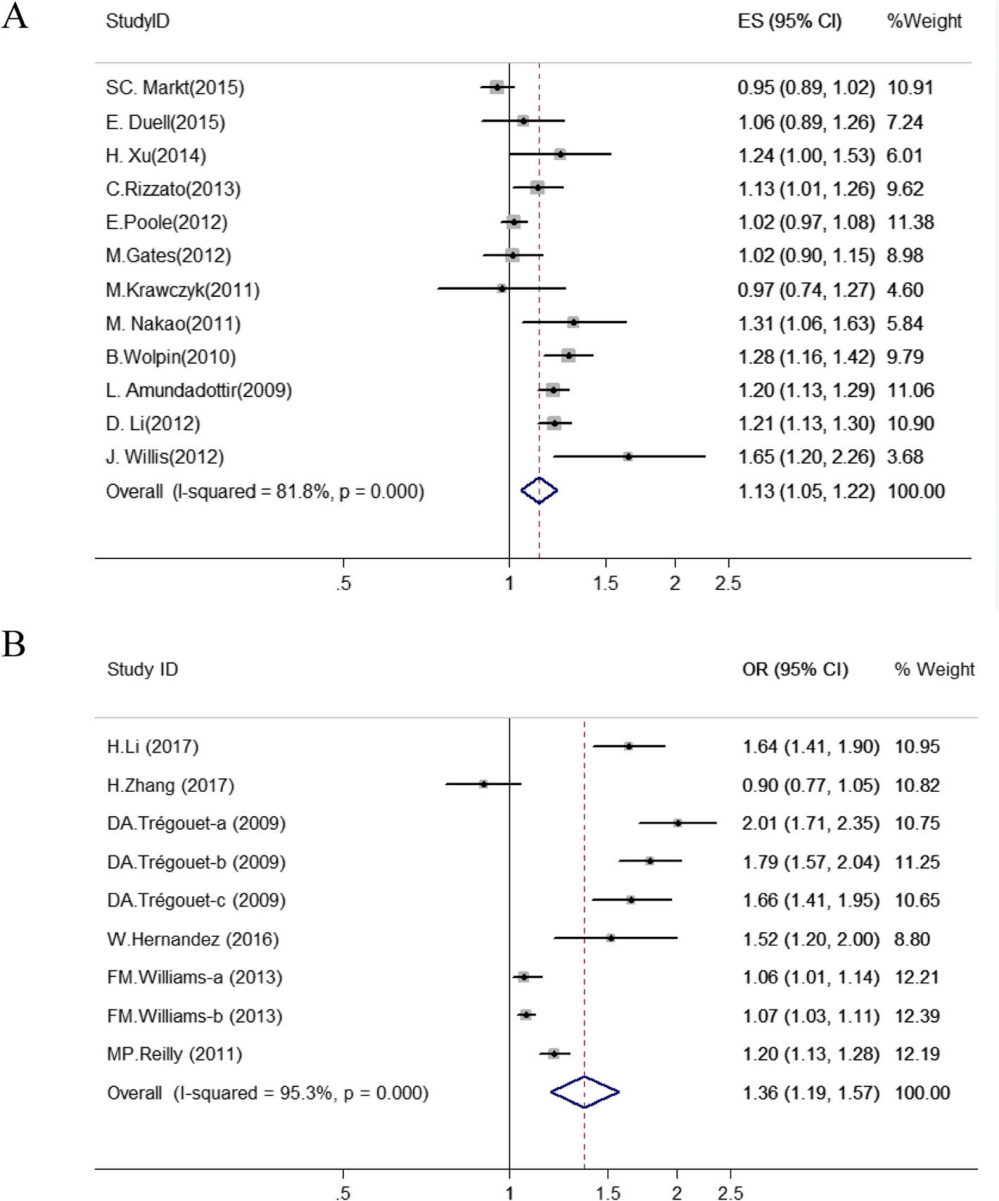
Forest plot of the relationship between rs505922 polymorphisms and cancer (**a**) and cardiocerebrovascular disease (**b**) risk (allele model and random-effect model). The circle and horizontal lines correspond to OR and 95% CI and the area of the squares reflects the weight of individual studies included in the meta-analysis. The diamond represents the pooled ORs and 95% CI. The dotted red line represents the total OR value

Meta-analysis was conducted to estimate the associations between rs505922 and cancer risk (Fig. 2a; Table S4) in 20,820 cancer cases and 27,837 controls. The rs505922 polymorphism was significantly associated with an increased cancers risk in the allele model (OR = 1.13, 95%CI = 1.05–1.22, *P* = 0.001). Subgroup analysis was conducted based on ethnicity, type of cancer, and source of control. The association between rs505922 and cancer risk was identified in Asian population subgroup (OR = 1.27, 95%CI = 1.10–1.48, *P* = 0.002), Mixed population subgroup (OR = 1.22, 95%CI = 1.17–1.27, *P* < 0.001), Pancreatic cancers subgroup (OR = 1.23, 95%CI = 1.16–1.31, *P* < 0.001), and Hospital based control groups (OR = 1.30, 95%CI = 1.12–1.51, *P* = 0.003) (Table S5; Fig. S1). However, no significant association was observed in Caucasian population(OR = 1.05, 95%CI = 0.97–1.13, *P* = 0.232). Sensitivity analyses were conducted by omitting each individual article to measure its specific effect on the pooled ORs (Fig. S2a). Sensitivity analysis plot indicated that no single study significantly affected the combined OR of SNP loci. Because of the heterogeneity of the research, we use the random effect model (allele model: *I* ^*2*^ = 81.8%) (Table S4). No significant publication bias was observed in any studies of SNPs (Fig. 4a; Table S4). After the applying the trim and fill method, there is no change in the OR value after the combination, also indicating that the original result is stable (Fig. S3).

We also performed a meta-analysis to evaluate the association between rs505922 SNP and cardiocerebrovascular diseases (Fig. 2b; Table S4). The rs505922 SNP was significantly associated with an increased cardiocerebrovascular diseases risk in the allele model (C/T: OR = 1.36, 95%CI = 1.19–1.57, *P* < 0.001). Subgroup analysis indicated that rs505922 was associated with a significantly higher risk of cardiocerebrovascular diseases in Caucasian population subgroup (OR = 1.39, 95%CI = 1.19–1.64, *P* < 0.001, allele model), African population subgroup(OR = 1.52, 95%CI = 1.18–1.96, *P* = 0.001), Hospital based control(OR = 1.14, 95%CI = 1.05–1.23, *P* = 0.003) and Population based control(OR = 1.54, 95%CI = 1.18–2.02, *P* = 0.002) (Table S5; Fig. S4). However, no significant association was observed in Asian subgroup (OR = 1.21, 95%CI = 0.67–2.19, *P* = 0.524) (Table S5; Fig. S4). Sensitivity analysis showed the results of this study were stable (Fig. S2b). There was no significant publication bias among the enrolled studies in Begg’s and Egger’s test (Fig. 4b; Table S4). After applying the trim and fill method, no new literature has been added, indicating that the result is stable (Fig. S5).

### Meta-analysis of rs657152 polymorphisms

**Fig. 3:**
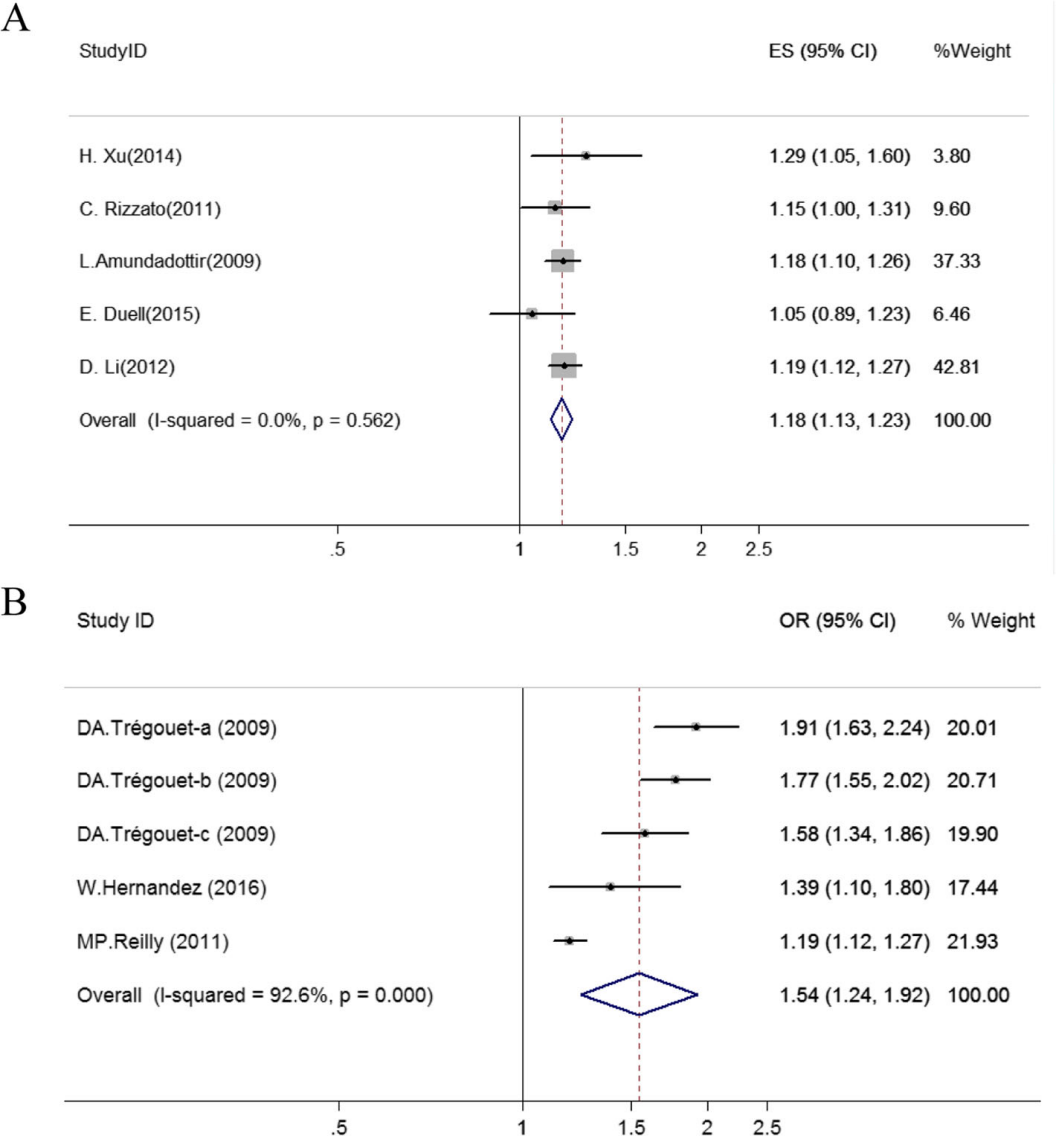
Forest plot of the relationship between rs657152 polymorphisms and cancer (**a**) and cardiocerebrovascular disease (**b**) risk (allele model and random-effect model). The circle and horizontal lines correspond to OR and 95% CI and the area of the squares reflects the weight of individual studies included in the meta-analysis. The diamond represents the pooled ORs and 95% CI. The dotted red line represents the total OR value

**Fig. 4:**
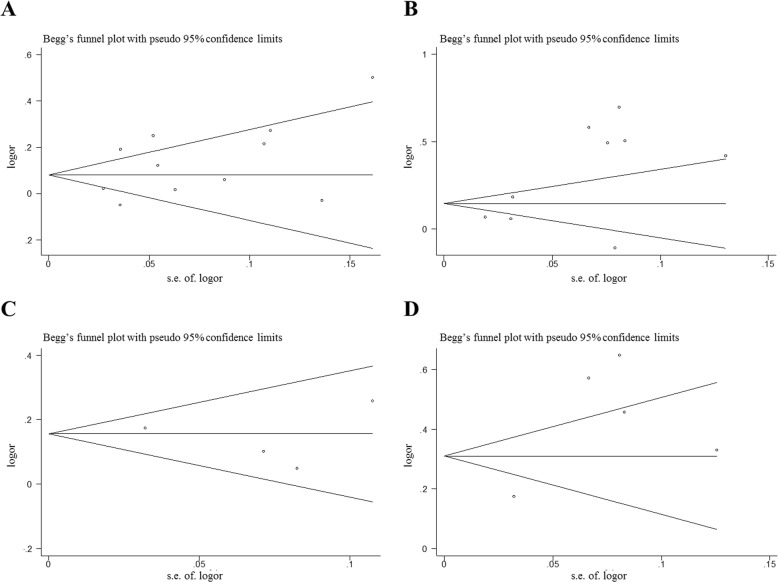
Begg’s funnel plot of publication bias test for rs505922 and rs657152 (allele model and random-effect model); Each point represents a separate study for the indicated association between rs505922 and cancer (**a**)/cardiocerebrovascular disease (**b**) risk, and rs657152 and cancer (**c**)/cardiocerebrovascular disease (**d**) risk, respectively. Each point stands for an individual article in overall population under allele model. s.e., standardized effect

Five studies reported the association between rs657152 and cancers risk. Our results showed that rs657152 was significantly associated with increased cancers risk in allele model (OR = 1.18, 95%CI = 1.13–1.23, *P* < 0.001) (Fig. 3a; Table S4). Sensitivity analysis revealed no significant influence on the pooled OR by any individual study (Fig. S6a). We conducted Begg’s and Egger’s tests to assess the publication bias for these studies and no evidence of publication bias was detected among the enrolled studies (Fig. 4c; Table S4). According to the results of trim and fill method, the result was stable (Fig. S7).

We also evaluated the relationship between rs657152 and cardiocerebrovascular diseases. A significant association between rs657152 and cardiocerebrovascular diseases under allele model were observed (OR = 1.54, 95%CI = 1.24–1.92, *P* < 0.001) (Fig. 3b; Table S4). Sensitivity analysis suggested that there was no significant change in the overall outcomes after removing any of the studies (Fig. S6b). The Begg’s and Egger’s tests also showed that no meaningful publication bias were found (Fig. 4d; Table S4). The result of the trim and fill method proves that the result was stable (Fig. S8).

## Discussion

In this study, we conducted a meta-analysis to clarify the relationship between ABO SNPs (rs505922 and rs657152) and cancer/cardiocerebrovascular diseases risk. Our results showed that these two SNPs were associated with pancreatic cancer risk and also increased the risk of cardiocerebrovascular diseases.

Regarding to rs505922 SNP, our results showed that the variant type of rs505922 could increase the risk of overall cancer, suggesting a potential predictive ability of this SNP for cancer risk. When we conducted a subgroup analysis of rs505922 based on cancer sites and ethnicity, we found that there was no significant association between rs505922 and Non-pancreatic cancer subgroup or Caucasian subgroup. This result may be due to heterogeneity of cancer types or insufficient statistical power. Therefore, further studies with large samples size are warrant to evaluate the association between the rs505922 polymorphisms in Non-pancreatic cancers. On the other side, our data also showed that rs505922 was associated with cardiocerebrovascular diseases. In subgroup analysis, we found that there was no association between rs505922 and cardiocerebrovascular diseases in Asian subgroup. Only two studies with small population were included in this analysis, further studies are needed in Asian population. The SNP is located in the first intron region of ABO gene, the protective T allele of rs505922 is in complete linkage disequilibrium (r^2^ = 1.0) with the O allele, is marks of O allele. However, the regulatory mechanism underlying the expression of histoblood group antigens was unclear.

For rs657152, our study demonstrated that this SNP was associated with cancer/ cardiocerebrovascular diseases risk. However, only eight studies [12–15, 17, 20, 30, 31] were reported and most of studies were conducted in Caucasian population. Therefore, more studies with different ethnic background and larger sample size are needed in the future. Rs657152 is located in the intron area of ABO, the possible function has not been revealed yet. Rs657152 has been found to be associated with several biological molecule, including LDL cholesterol [36], liver derived alkaline phosphatas [37], and IL-6 [38]. This implied that rs657152 may affect the occurrence and development of disease by influencing these biological molecule. Further subsequent functional studies are warrant.

The underlying mechanism for the relationship between ABO blood group and cancer risk is still poorly understood [39]. It is reported that blood type may affect the progression and expansion of malignant tumors by altering the systemic inflammatory response [40]. Recent studies reported an association between polymorphisms at the ABO gene locus and circulating levels of tumour necrosis factor-alpha [41], soluble intercellular adhesion molecule (ICAM)-1 [42, 43], E-selectin [44, 45], and P-selectin [43]. These adhesion molecules were important mediators of chronic inflammation and immune cell recruitment [46]. They may provide a biological basis for the postulated influence of ABO on cancer survival, by linking ABO blood group and tumour initiation and spread [39]. In addition, some researches have shown that the structure of certain tumor antigens was similar to the structure of antigens of ABO blood group system. Smith and Prieto [47] suggested the Forssmann antigen which predominant in stomach and colon tumors, was almost structurally identical to the A antigen determinant. Blood group A carrier may have diminished tumor immune response due to reduced ability to recognize and attack tumor cells [48].

There was some evidence linking ABO blood group and cardiocerebrovascular diseases. Jenkins, P.V. et al. reported an association between ABO blood types and von Willebrand factor(vWF) and factor VIII(FVIII), both of which play crucial roles in the coagulation pathway [49]. Higher levels of vWF and FVIII has been observed in non-O blood type than O blood type [50]. Therefore, type O blood may be a risk factor for bleeding [51]. In addition, the non-O blood group has been shown to be correlated with higher total cholesterol and LDL-C levels [52], and the latest study proposed that approximately 10% of the effect of ABO blood group on coronary artery disease (CAD) susceptibility was mediated by plasma cholesterol levels [53].

Limitations in this study should be mentioned. First, the studies included in our meta-analysis were limited to published reports and English language studies. Unpublished reports or those published in non-English language studies were not included in the analysis. It would limit our sample size and publication bias might be exist. Second, both of the hospital based and population based case-control studies were included in our study. Therefore, selection bias would be exist compared to the meta-analysis only included population based case-control studies. Third, the limited number of published studies may influence the reliability of our results. Finally, the lack of original data limited further evaluations of the potential gene-gene and gene-environment interactions.

## Conclusion

In summary, the results of our meta-analysis revealed that rs505922 and rs657152 were correlated with an increased pancreatic cancers risk. Due to most of the studies were conducted in pancreatic cancer type and Caucasian populations, further studies in multiple cancer types and multiple ethnic populations are needed. In addition, our meta-analysis also revealed that rs505922 and rs657152 associated with cardiocerebrovascular diseases. However, owing to limited number of studies, further studies with larger samples size are warrant. This study can provide clues for further exploration of novel biomarkers with cancer/cardiocerebrovascular early-warning function.

## Supplementary information

**Additional file 1. Figure S1** Subgroup analysis of rs505922 and cancer risk. **Figure S2** Sensitivity analysis diagram of rs505922 and cancer (a) and cardiocerebrovascular disease (b) risk. **Figure S3** Trim and fill method results (a) and filled funnel plot (b) of rs505922 and cancer risk. **Figure S4** Subgroup analysis of rs505922 and cardiocerebrovascular disease risk. **Figure S5** Trim and fill method results (a) and filled funnel plot (b) of rs505922 and cardiocerebrovascular disease risk. **Figure S6** Sensitivity analysis of rs657152 and cancer (a) and cardiocerebrovascular disease (b) risk. **Figure S7** Trim and fill method results (a) and filled funnel plot (b) of rs657152 and cancer risk. **Figure S8** Trim and fill method results (a) and filled funnel plot (b) of rs657152 and cardiocerebrovascular disease risk. **Table S1**. Previous reported SNPs had strong linkage disequilibrium with rs505922. **Table S2** Quality assessment of case-control studies according to NOS for rs505922. **Table S3** Quality assessment of case-control studies according to NOS for rs657152. **Table S4** Results for meta-analysis of ABO gene polymorphisms with cancer and cardiocerebrovascular disease risk. **Table S5** Subgroup analysis of rs505922 polymorphism

## Data Availability

Not applicable.

## Abbreviations

HB: Hospital based
NOS: Newcastle-Ottawa Scale
PB: Population based
