# Supplementary material for: Association of ABO polymorphisms and pancreatic Cancer/ Cardiocerebrovascular disease: a meta-analysis
Source: BMC Med Genet. 2020 Feb 24;21:41. doi: 10.1186/s12881-020-0975-8 (PMC7041112; doi:10.1186/s12881-020-0975-8)
Supplement: Supplementary file 1 — Additional file 1. Figure S1 Subgroup analysis of rs505922 and cancer risk. Figure S2 Sensitivity analysis diagram of rs505922 and cancer (a) and cardiocerebrovascular disease (b) risk. Figure S3 Trim and fill method results (a) and filled funnel plot (b) of rs505922 and cancer risk. Figure S4 Subgroup analysis of rs505922 and cardiocerebrovascular disease risk. Figure S5 Trim and fill method results (a) and filled funnel plot (b) of rs505922 and cardiocerebrovascular disease risk. Figure S6 Sensitivity analysis of rs657152 and cancer (a) and cardiocerebrovascular disease (b) risk. Figure S7 Trim and fill method results (a) and filled funnel plot (b) of rs657152 and cancer risk. Figure S8 Trim and fill method results (a) and filled funnel plot (b) of rs657152 and cardiocerebrovascular disease risk. Table S1. Previous reported SNPs had strong linkage disequilibrium with rs505922. Table S2 Quality assessment of case-control studies according to NOS for rs505922. Table S3 Quality assessment of case-control studies according to NOS for rs657152. Table S4 Results for meta-analysis of ABO gene polymorphisms with cancer and cardiocerebrovascular disease risk. Table S5 Subgroup analysis of rs505922 polymorphism [file 12881_2020_975_MOESM1_ESM.doc]

**Association of ABO Polymorphisms and Cancer/****Cardiocerebrovascular Disease: a Meta-analysis.**

Yanxia Li*, Luyang Liu*, Yubei Huang, Hong Zheng, Lian Li

Department of Epidemiology and Biostatistics, National Clinical Research Center for Cancer, Key Laboratory of Molecular Cancer Epidemiology of Tianjin, Tianjin Medical University Cancer Institute and Hospital, Tianjin, China.

* These authors contributed equally to this work.

**Correspondence to**:

Lian Li ([lilian@tmu.edu.cn](mailto:lilian@tmu.edu.cn)) or Hong Zheng ([zhengh64@aliyun.com](mailto:zhengh64@aliyun.com) )

**Supplemental Figure Legends**

Figure S1 Subgroup analysis of rs505922 and cancer risk.a: Ethnicity; b: Source of control; c: Cancer stype. The circle and horizontal lines correspond to OR and 95% CI and the area of the squares reflects the weight of individual studies included in the meta-analysis. The diamond represents the pooled ORs and 95% CI. The dotted red line represents the total OR value.

Figure S2 Sensitivity analysis diagram of rs505922 and cancer (a) and cardiocerebrovascular disease (b) risk.

Figure S3 Trim and fill method results (a) and filled funnel plot (b) of rs505922 and cancer risk

Figure S4 Subgroup analysis of rs505922 and cardiocerebrovascular disease risk. a: Ethnicity; b: Source of control. The circle and horizontal lines correspond to OR and 95% CI and the area of the squares reflects the weight of individual studies included in the meta-analysis. The diamond represents the pooled ORs and 95% CI. The dotted red line represents the total OR value.

Figure S5 Trim and fill method results (a) and filled funnel plot (b) of rs505922 and cardiocerebrovascular disease risk

Figure S6 Sensitivity analysis of rs657152 and cancer (a) and cardiocerebrovascular disease (b) risk

Figure S7 Trim and fill method results (a) and filled funnel plot (b) of rs657152 and cancer risk

Figure S8 Trim and fill method results (a) and filled funnel plot (b) of rs657152 and cardiocerebrovascular disease risk

**Supplemental Tables**

Table S1. Previous reported SNPs had strong linkage disequilibrium with rs505922

| **SNP loci** | **Year** | **First author** | **Ethnicity** | **Cancer/disease** | **Control*** | **Sample size**  **(case/control)** | **Case** | | |  | **Control** | | | **OR(95%CI)** |
| --- | --- | --- | --- | --- | --- | --- | --- | --- | --- | --- | --- | --- | --- | --- |
|  |  |  |  |  |  |  | **Hom**^†^ **Het**^†^ **Hom**  **wild variant** | | |  | **Hom Het Hom**  **wild variant** | | |  |
| rs529565 | 2017 | H. Li | Asian | Ischemic stroke | PB | 991/1002 | 521 | 383 | 87 |  | 578 | 356 | 68 | 1.20(1.04-1.38) |
|  | 2016 | XM. Ling | Asian | Ischemic stroke | PB | 883/955 | 343 | 401 | 139 |  | 390 | 421 | 144 | 1.06 (0.90-1.24) |
|  | 2016 | DA. Hinds | Caucasian | Thrombotic disease | PB | 6135/252827 | -- | -- | -- |  | -- | -- | -- | 1.15 (1.13-1.17) |
|  | 2016 | XM. Ling | Asian | Large-artery  atherosclerosis | PB | 918/979 | 151 | 216 | 79 |  | 390 | 421 | 144 | 1.24 (1.02-1.51) |
|  | 2015 | M. Germain | Caucasian | Venous thromboembolism | PB/HB | 7507/52632 | -- | -- | -- |  | -- | -- | -- | 1.55 (1.48–1.63) |
| rs643434 | 2011 | J A. Heit | Caucasian | Venous thromboembolism | PB | 1488/1439 | -- | -- | -- |  | -- | -- | -- | 1.44 (1.30–1.61) |
| rs514659 | 2016 | XM. Ling | Asian | Coronary artery disease | PB | 918/979 | -- | -- | -- |  | -- | -- | -- | 1.21(1.13-1.30) |
| rs514659 | 2015 | M. Bruzelius | Caucasian | Venous thromboembolism | PB | 1425/1350 | -- | -- | -- |  | -- | -- | -- | 1.51 (1.35–1.69) |
|  | 2011 | M P. Reilly | Caucasian | Myocardial infarction | HB | 5783/3644 | -- | -- | -- |  | -- | -- | -- | 1.21(1.13–1.29) |
| rs687289 | 2011 | M P. Reilly | Caucasian | Myocardial infarction | HB | 5783/3644 | -- | -- | -- |  | -- | -- | -- | 1.20 (1.13–1.29) |
|  | 2011 | J A.Heit | Caucasian | Venous thromboembolism | PB | 1488/1439 | -- | -- | -- |  | -- | -- | -- | 1.48 (1.33–1.65) |
| rs687621 | 2016 | W. Hernandez | Caucasian | Venous thrombosis | HB | 146 /432 | -- | -- | -- |  | -- | -- | -- | 1.55 (1.2-2.0) |
|  | 2011 | M P. Reilly | Caucasian | Myocardial infarction | HB | 5783/3644 | -- | -- | -- |  | -- | -- | -- | 1.20 (1.13–1.28) |

*PB: population based control, HB: hospital based control; †Hom: homozygous, Het: heterozygote

Table S2 Quality assessment of case-control studies according to NOS for rs505922

| Study ID | Authors | Years | | Selection | | | | Comparability | | Exposure | | | Total score |
| --- | --- | --- | --- | --- | --- | --- | --- | --- | --- | --- | --- | --- | --- |
|  |  |  |  | A | B | C | D | E | F | G | H | I |  |
| 1 | H. Xu | 2014 | | 1 | 1 | 1 | 1 | 1 | 0 | 1 | 1 | 1 | 8 |
| 2 | E. Duell | 2015 | | 1 | 1 | 1 | 0 | 1 | 1 | 1 | 1 | 1 | 8 |
| 3 | C.Rizzato | 2013 | | 1 | 1 | 1 | 0 | 1 | 1 | 1 | 1 | 1 | 8 |
| 4 | E.Poole | 2012 | | 1 | 1 | 1 | 0 | 0 | 1 | 1 | 1 | 1 | 7 |
| 5 | M.Krawczyk | 2011 | | 1 | 1 | 1 | 1 | 1 | 0 | 1 | 1 | 1 | 9 |
| 6 | M. Nakao | 2011 | | 1 | 1 | 0 | 1 | 0 | 1 | 1 | 1 | 1 | 8 |
| 7 | M.Gates | 2012 | | 1 | 1 | 1 | 0 | 0 | 0 | 1 | 1 | 1 | 7 |
| 8 | B.Wolpin | 2010 | | 1 | 1 | 0 | 1 | 1 | 1 | 1 | 1 | 1 | 9 |
| 9 | D. Li | 2012 | | 1 | 1 | 0 | 1 | 1 | 1 | 1 | 1 | 1 | 8 |
| 10 | J. Willis | 2012 | | 1 | 1 | 0 | 1 | 1 | 1 | 1 | 1 | 1 | 8 |
| 11 | SC. Markt | 2015 | | 1 | 1 | 0 | 1 | 1 | 1 | 1 | 0 | 1 | 7 |
| 12 | H.Li | 2017 | | 1 | 1 | 0 | 1 | 1 | 1 | 1 | 1 | 1 | 8 |
| 13 | FM.Williams | 2013 | | 1 | 1 | 0 | 0 | 0 | 0 | 1 | 1 | 1 | 6 |
| 14 | H. Zhang | 2017 | | 1 | 1 | 1 | 1 | 0 | 1 | 1 | 1 | 1 | 8 |
| 15 | DA. Tregouët | | 2009 | 1 | 1 | 1 | 1 | 0 | 0 | 1 | 1 | 1 | 7 |
| 16 | L.Amundadottir | 2009 | | 1 | 1 | 0 | 1 | 1 | 1 | 1 | 1 | 0 | 8 |
| 17 | W.Hernandez | 2016 | | 1 | 1 | 0 | 1 | 0 | 0 | 1 | 1 | 1 | 6 |
| 18 | MP. Reilly | 2011 | | 1 | 1 | 0 | 1 | 1 | 1 | 1 | 1 | 1 | 8 |

Selection: A: Is the case definition adequate? B: Representativeness of the cases, C: Selection of controls, D: Definition of controls; Comparability: E: Study controls for age and gender, F: Study controls for any additional factor; Exposure: G: Ascertainment of exposure, H: Same method of ascertainment for cases and controls, I: Non-response rate

Table S3 Quality assessment of case-control studies according to NOS for rs657152

| Study ID | Authors | Years | Selection | | | | Comparability | | Exposure | | | Total score |
| --- | --- | --- | --- | --- | --- | --- | --- | --- | --- | --- | --- | --- |
|  |  |  | A | B | C | D | E | F | G | H | I |  |
| 1 | C.Rizzato | 2011 | 1 | 1 | 1 | 0 | 0 | 1 | 1 | 1 | 1 | 7 |
| 2 | H. Xu | 2014 | 1 | 1 | 1 | 1 | 1 | 0 | 1 | 1 | 1 | 8 |
| 3 | E. Duell | 2015 | 1 | 1 | 1 | 0 | 1 | 1 | 1 | 1 | 1 | 8 |
| 4 | D.Li | 2012 | 1 | 1 | 0 | 1 | 1 | 1 | 1 | 1 | 1 | 8 |
| 5 | DA.Tregouët | 2009 | 1 | 1 | 1 | 1 | 0 | 0 | 1 | 1 | 1 | 7 |
| 6 | W. Hernandez | 2016 | 1 | 1 | 0 | 1 | 0 | 0 | 1 | 1 | 1 | 6 |
| 7 | M P. Reilly | 2011 | 1 | 1 | 0 | 1 | 1 | 1 | 1 | 1 | 1 | 8 |
| 8 | L.Amundadottir | 2009 | 1 | 1 | 0 | 1 | 1 | 1 | 1 | 1 | 0 | 8 |

Selection: A: Is the case definition adequate? B: Representativeness of the cases, C: Selection of controls, D: Definition of controls; Comparability: E: Study controls for age and gender, F: Study controls for any additional factor; Exposure: G: Ascertainment of exposure, H: Same method of ascertainment for cases and controls, I: Non-response rate

Table S4 Results for meta-analysis of ABO gene polymorphisms with cancer and [cardiocerebrovascular disease](../../../../C:/Program%20Files%20(x86)/Youdao/Dict/7.5.2.0/resultui/dict/javascript:%3B) risk

|  | No. | OR(95%CI) | P-Value | Heterogeneity | | Publication bias | |
| --- | --- | --- | --- | --- | --- | --- | --- |
|  |  |  |  | I^2^ | P_Q_ | Egger’s test | Begg’s test |
| **Cancer** |  |  |  |  |  |  |  |
| rs505922(T>C) |  |  |  |  |  |  |  |
| C compared with T allele | 12 | 1.13(1.05-1.22 ) | 0.001 | 81.8% | <0.001 | 0.330 | 0.732 |
| rs657152(G>A) |  |  |  |  |  |  |  |
| A compared with G allele | 5 | 1.18(1.13-1.23) | <0.001 | 0.0% | 0.562 | 0.676 | 0.806 |
| [**Cardiocerebrovascular disease**](../../../../C:/Program%20Files%20(x86)/Youdao/Dict/7.5.2.0/resultui/dict/javascript:%3B) |  |  |  |  |  |  |  |
| rs505922(T>C) |  |  |  |  |  |  |  |
| C compared with T allele | 9 | 1.36(1.19-1.57) | <0.001 | 95.3% | <0.001 | 0.031 | 0.211 |
| rs657152(G>A) |  |  |  |  |  |  |  |
| A compared with G allele | 5 | 1.54(1.24-1.92) | <0.001 | 92.6% | <0.001 | 0.132 | 1.00 |

Table S5 Subgroup analysis of rs505922 polymorphism

|  | No. | Allele Model | | | | Sample size | |
| --- | --- | --- | --- | --- | --- | --- | --- |
|  |  | OR(95%CI) | p | I^2^ | P_Q_ |  |  |
| **Cancer** |  |  |  |  |  |  | |
| Overall | 12 | 1.13(1.05-1.22) | 0.001 | 81.8% | <0.001 | 20820/27837 | |
| Ethnicity |  |  |  |  |  |  | |
| Caucasian | 7 | 1.05(0.97-1.13) | 0.232 | 63.8% | 0.011 | 11103/16411 | |
| Asian | 2 | 1.27(1.10-1.48) | 0.002 | 0.0% | 0.704 | 441/2013 | |
| Mixed | 3 | 1.22(1.17-1.27) | <0.001 | 0.0% | 0.558 | 9276/9449 | |
| Cancer sites |  |  |  |  |  |  | |
| Pancreatic cancer | 7 | 1.22(1.27-1.28) | <0.001 | 16.4% | 0.305 | 11130/13868 | |
| Other sites | 5 | 1.00(0.96-1.04) | 0.983 | 0.0% | 0.525 | 9690/14005 | |
| Source of control |  |  |  |  |  |  | |
| HB | 3 | 1.30(1.12-1.51) | 0.003 | 48.1% | 0.145 | 4421/5548 | |
| PB | 5 | 1.12(0.99-1.26) | 0.062 | 64.1% | 0.025 | 3473/4855 | |
| HB/PB | 3 | 1.11(0.99-1.25) | 0.063 | 86.7% | 0.001 | 10152/13027 | |
| [**Cardiocerebrovascular disease**](../../../../C:/Program%20Files%20(x86)/Youdao/Dict/7.5.2.0/resultui/dict/javascript:%3B) | | | | | | | |
| Overall | 9 | 1.36(1.19-1.57) | <0.001 | 95.3% | <0.001 | | 22275/71549 |
| Ethnicity |  |  |  |  |  | |  |
| Caucasian | 6 | 1.39(1.19-1.64) | <0.001 | 96.2% | <0.001 | | 20494/69473 |
| Asian | 2 | 1.21(0.67-2.19) | 0.524 | 96.7% | <0.001 | | 1635/1644 |
| African | 1 | 1.52(1.18-1.96) | 0.001 | - | - | | 146/432 |
| Source of control* |  |  |  |  |  | |  |
| HB | 4 | 1.14(1.05-1.23) | 0.003 | 82.5% | <0.001 | | 18464/67269 |
| PB | 5 | 1.54(1.18-2.02) | 0.002 | 93.7% | <0.001 | | 3811/4280 |

*PB: population based control, HB: hospital based control

**Supplemental Figures**

**Figure S1**


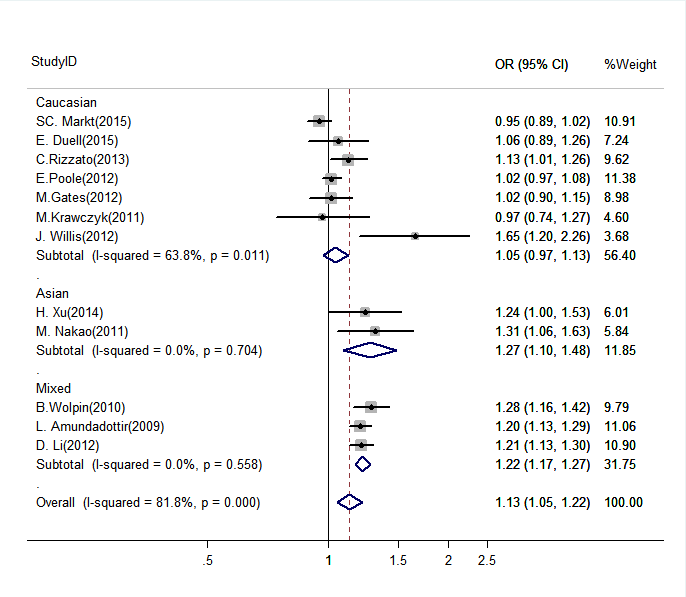


**A**


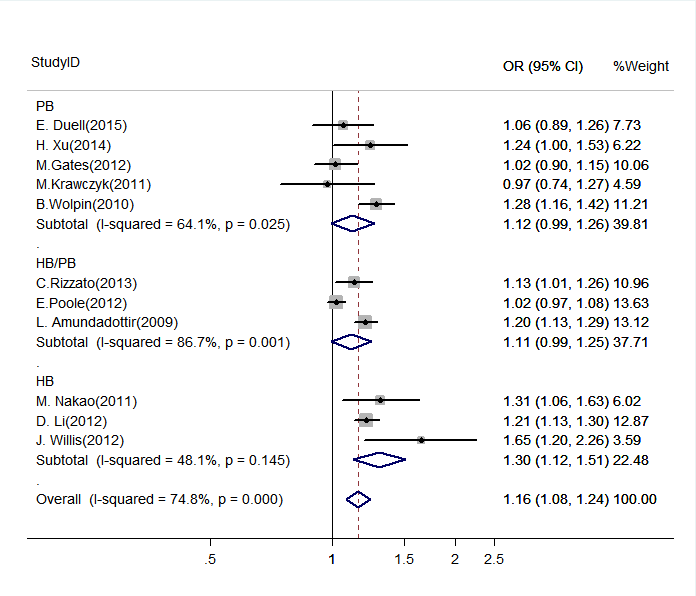


**B**


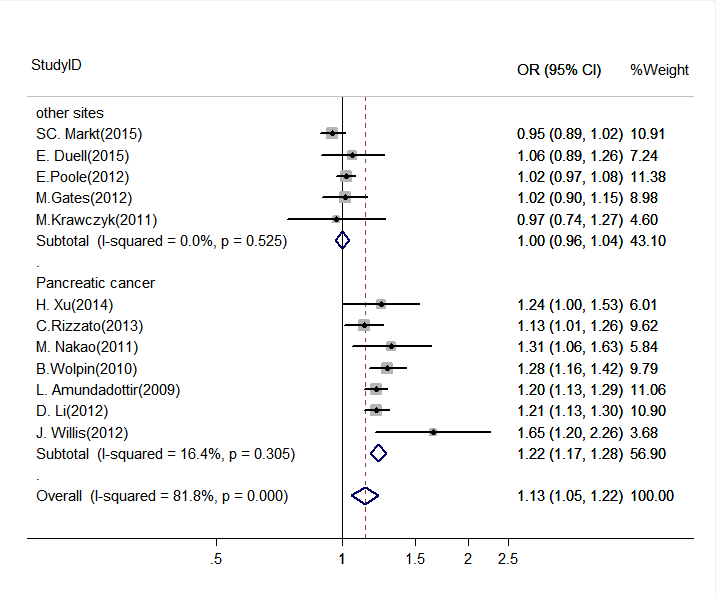


**C**

**Figure S2**

**A**

**B**

**Figure S3**

**A**

**A**

| Method | Pooled  Est | 95% CI | | Asymptotic | | No. of  studies |
| --- | --- | --- | --- | --- | --- | --- |
|  |  | Lower | Upper | z-value | p-value |  |
| Fixed | 1.104 | 1.074 | 1.134 | 7.106 | 0.000 | 12 |
| Random | 1.134 | 1.055 | 1.220 | 3.397 | 0.001 |  |

**B**

**Figure S4**


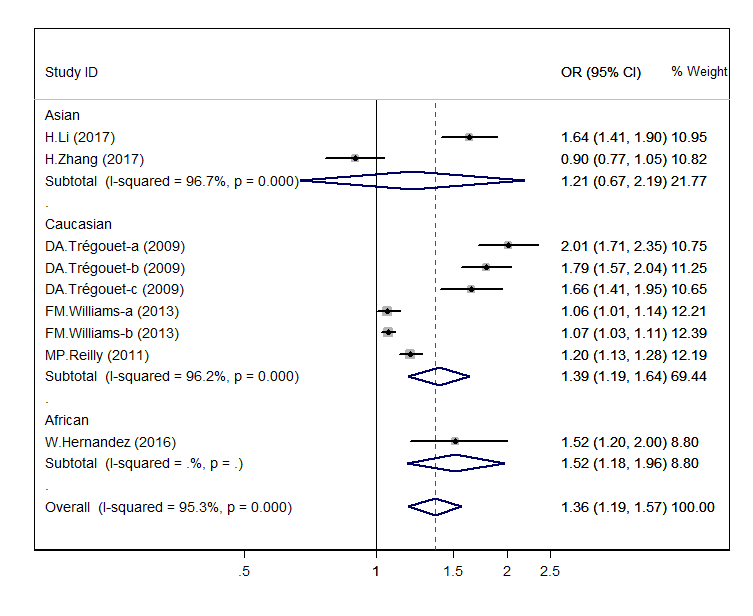


A


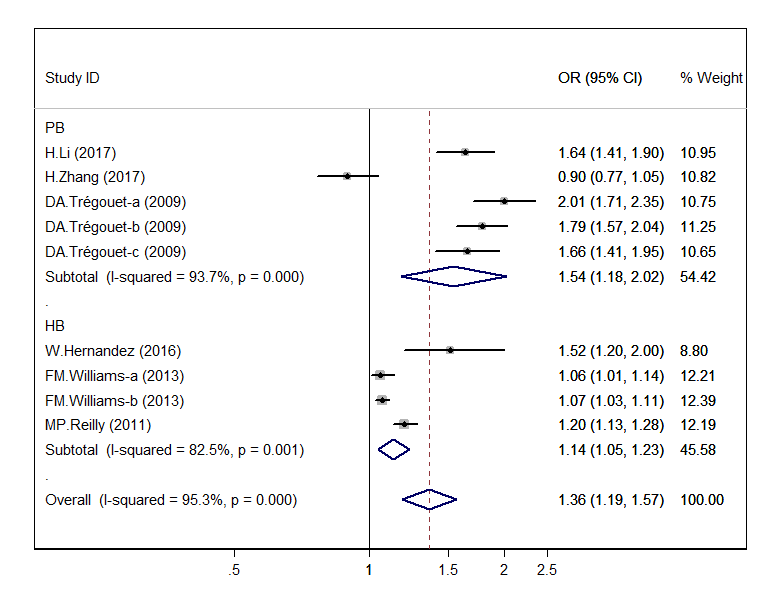


**B**

**Figure S5**

**A**

| Method | Pooled | 95% CI | | Asymptotic | | No. of  studies |
| --- | --- | --- | --- | --- | --- | --- |
|  | Est | Lower | Upper | z_value | p_value |
| Fixed | 1.156 | 1.127 | 1.187 | 10.972 | 0.000 | 9 |
| Random | 1.364 | 1.187 | 1.568 | 4.371 | 0.000 |

**B**

**Figure S6**

**A**

**B**

**Figure S7**

**A**

| Method | Pooled  Est | 95% CI | | Asymptotic | | No. of  studies |
| --- | --- | --- | --- | --- | --- | --- |
|  |  | Lower | Upper | z_value | p_value |  |
| Fixed | 1.177 | 1.129 | 1.226 | 7.748 | 0.000 | 5 |
| Random | 1.177 | 1.129 | 1.226 | 7.748 | 0.000 |  |

**B**

**Figure S8**

**A**

| Method | Pooled | 95% CI | | Asymptotic | | No. of  studies |
| --- | --- | --- | --- | --- | --- | --- |
|  | Est | Lower | Upper | z_value | p_value |
| Fixed | 1.364 | 1.298 | 1.434 | 12.277 | 0.000 | 5 |
| Random | 1.544 | 1.240 | 1.924 | 3.876 | 0.000 |  |

**B**
